# Robust Spacecraft Component Detection in Point Clouds

**DOI:** 10.3390/s18040933

**Published:** 2018-03-21

**Authors:** Quanmao Wei, Zhiguo Jiang, Haopeng Zhang

**Affiliations:** 1 Image Processing Center, School of Astronautics, Beihang University, Beijing 100191, China; weiqm@buaa.edu.cn (Q.W.); jiangzg@buaa.edu.cn (Z.J.); 2 Beijing Key Laboratory of Digital Media, Beijing 100191, China

**Keywords:** geometric primitive, component detection, spacecraft, 3D point clouds

## Abstract

Automatic component detection of spacecraft can assist in on-orbit operation and space situational awareness. Spacecraft are generally composed of solar panels and cuboidal or cylindrical modules. These components can be simply represented by geometric primitives like plane, cuboid and cylinder. Based on this prior, we propose a robust automatic detection scheme to automatically detect such basic components of spacecraft in three-dimensional (3D) point clouds. In the proposed scheme, cylinders are first detected in the iteration of the energy-based geometric model fitting and cylinder parameter estimation. Then, planes are detected by Hough transform and further described as bounded patches with their minimum bounding rectangles. Finally, the cuboids are detected with pair-wise geometry relations from the detected patches. After successive detection of cylinders, planar patches and cuboids, a mid-level geometry representation of the spacecraft can be delivered. We tested the proposed component detection scheme on spacecraft 3D point clouds synthesized by computer-aided design (CAD) models and those recovered by image-based reconstruction, respectively. Experimental results illustrate that the proposed scheme can detect the basic geometric components effectively and has fine robustness against noise and point distribution density.

## 1. Introduction

Automatic tracking of space objects, recognizing them and estimating their relative poses are the main tasks in space exploitation and space-based space surveillance [1,2,3,4,5]. Recent advances in three-dimensional (3D) data acquisition have resulted in a broad availability of 3D data [6]. The 3D models of space objects have obvious benefits in space applications such as pose estimation, autonomous rendezvous and docking, on-orbit servicing and active debris removal [7,8,9,10], especially for non-cooperative spacecraft without cooperative markers. Comparing to 2D images, the 3D data are free of perspective projection and can reveal the structural information of objects in 3D space, such as shape, dimension, position and orientation. Unstructured point cloud data are a popular kind of 3D data, which can be generated by directly scanning with 3D sensors or image-based reconstruction techniques such as simultaneous localization and mapping (SLAM) [11,12] and structure from motion (SFM) [13,14]. However, to use the point cloud data for higher level tasks, such as on-orbit operation and situational awareness, further processing is still required in order to extract meaningful abstract information of the recorded object.

Recognition of patterns in an image is a rather easy task for a human; however, it is really hard for a computer. Such a fact facilitates studies in the fields of image processing and computer vision. The same case occurs in understanding 3D point clouds. For most of the 3D data processing and analyzing approaches, detection of geometric primitives is a crucial procedure [15,16,17]. For example, to generate 3D models of city buildings with LiDAR data, plane detection is generally employed for rooftop segmentation [18,19]. While to model installations in industrial sites where pipes are frequently encountered, cylinders are usually detected [20,21,22]. Hough transform (HT) [23], random sample consensus (RANSAC) [24,25] and surface growing [19] are frequently used for 3D plane and cylinder detection in most of these approaches. Plane detection by HT is a natural extension of the 2D Hough transform for line detection in images. Due to directly parameterizing five freedoms for a cylinder could lead to large memory and computational consumptions in HT, cylinder detection is usually divided into detection of cylinder axes and estimation of radius and position [20]. Besides, model fitting is an enabling alternative, where the shapes to be detected are treated as undetermined models [26,27] and finally detected along with supports by model fitting. The work in [26] formulates geometric multi-model fitting as an optimal labeling problem, in which the global energy function can be minimized by an extension version [28] of the α-expansion algorithm [29]. Then, multi-models are fitted in the iteration of the proposed expanding and re-estimating labels (PEARL). Such an energy-based multi-model fitting method is successfully used for plane detection in [27].

Most of these 3D data processing frameworks are interested in the modeling of city buildings and industrial installations, while little effort is made for other targets, such as spacecraft. As for the 3D data of spacecraft, feature points and local descriptors or global descriptors are often used for applications such as pose estimation and tracking, while further structure analysis is rarely involved, which is however crucial for future on-orbit operation and situational awareness, especially for non-cooperative space targets. Spacecraft mainly consist of solar panels, cuboid or cylinder modules; thus, they can also be regarded as a combination of geometric primitives like planes, cuboids or cylinders. Based on this simplification, a structural representation with higher level abstractions can be generated via the detection of such geometric primitives. Moreover, most of the 3D data processing approaches deal with laser scanning data, metric information and controllable quality, which are usually available; while this paper focuses on 3D data of spacecraft recovered by reconstruction, since it has more potential than laser scanning for the sake of hardware complexity, size and power requirement. Generally, such recovered data are often a scaled reconstruction rather than a metric one, and these data might also have different densities and noise levels due to variant texture quality. Therefore, special attention must be paid to scale variation, distribution variation and severe noise and outliers.

To address the automatic component detection for spacecraft, a robust detection scheme is developed in this paper, where components, such as solar panels, cuboid and cylinder modules, are detected as geometric primitives:

(1) Cylinders are first detected by PEARL [26], where initial cylinder proposals are generated in RANSAC, and energy-based multi-cylinder fitting and parameter estimation via least square fitting are then iteratively executed to detect the desired cylinders.
(2) Planes are detected using Hough transform and finally represented as bounded patches with their minimum bounding rectangles (MBR).
(3) Cuboids are recognized with pair-wise geometry relations from the detected patches at last.

A concise and abstract mid-level geometry representation of the spacecraft can be finally delivered. The performance of the proposed scheme is tested with synthesized point cloud data of eight spacecraft and the more challenging reconstructed point cloud data of 10 spacecraft. This paper is a further improvement of previous work [30]. Thus, we use the results of [30] as a baseline for comparison. Results on synthesized and reconstructed data are both promising. The contributions of this work are two-fold.

(1) The cuboid is a common geometry primitive, which can however not be parameterized directly. Detection and recognition of cuboids are still open problems. In this paper, we propose a new method to recognize cuboids from rectangle patches using geometry relation criteria to infer opposite and adjacent cuboid faces. Moreover, robust estimation methods for cuboid orientation and dimension are also proposed.
(2) We propose an entire automatic spacecraft component detection scheme, which can provide concise and abstract geometric representations of the space objects from unstructured 3D point cloud data. To make the scheme robust, we use some special procedures and improvements, such as the use of adaptive dimensional unit ε, utilization of PEARL for robust cylinder detection and improvement on MBR estimation.

The rest of this paper is organized as follows. Section 2 gives a detailed description of the proposed component detection scheme. Experimental results are presented in Section 3. Section 4 provides the conclusion.

## 2. The Proposed Scheme

**Figure 1:**
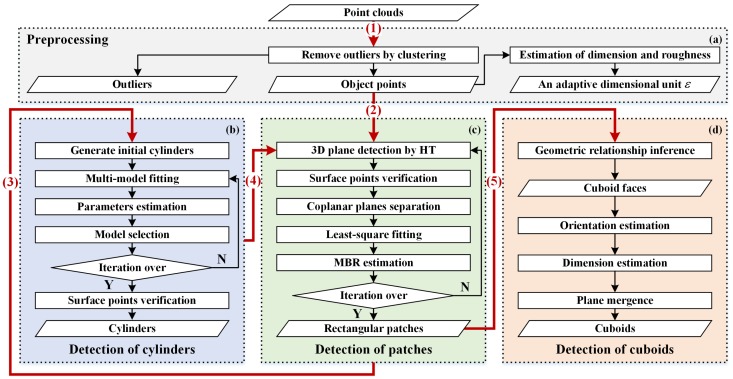
Procedure of the proposed spacecraft component detection scheme. The scheme consists of four modules: (**a**) preprocessing; (**b**) detection of cylinders; (**c**) detection of planar patches and (**d**) detection of cuboids. The scheme is implemented in the order from (1) to (5). Note that the flow lines starting with the borders of module blocks indicate that the remaining points are taken as input. HT, Hough transform; MBR, minimum bounding rectangles.

To automatically detect components of spacecraft in 3D point clouds, this paper develops a robust scheme, where cylinders, planar patches and cuboids can be successively detected. The procedure of the proposed scheme is shown in Figure 1. The scheme consists of four modules, namely preprocessing (Figure 1a), detection of cylinders (Figure 1b), planar patches (Figure 1c) and cuboids (Figure 1d). The preprocessing module is aimed at removing outliers and giving a relative dimensional unit ε, and the detection modules are designed to detect the corresponding geometric primitives.

The scheme is implemented with five steps:

(1) First, point cloud preprocessing is conducted to remove outliers. Meanwhile, an adjective dimensional unit ε is also estimated, which is useful in the subsequent detection steps.
(2) Since local areas of the cylinder surface can be regarded as planes in approximation and a plane can be also treated as a local area of a cylinder with a large radius, the existence of plane points and cylinder points will influence the detection of each other. Thus, to avoid the influence of plane points on cylinder detections, prominent patches (i.e., the top 10 planes with the most points in our cases) are removed in advance before cylinder detection via the patches detection module. Note that the actual patch detection is performed after cylinder detection, i.e., the next Steps (3) and (4).
(3) Cylinders are detected by the cylinder detection module in the fashion of PEARL, where initial cylinder proposals are first generated in RANSAC, then energy-based multi-cylinder fitting and parameter estimation via least square fitting are iteratively executed to detect the desired cylinder primitives. The surface points of these cylinder primitives are verified at last. Note that plane detection results of Step (2) will be revoked after primitives detection and before point verification.
(4) Planar patches are detected by the patches detection module, where the Hough transform is mainly employed. MBRs of these patches are estimated for a further representation.
(5) Cuboids are recognized by the cuboid detection module from the patches results of Step (4). Geometric relation criteria are first proposed to infer opposite and adjacent cuboid faces, through which patches belonging to the same cuboid could be detected. Then, the cuboid orientation and dimension are robustly estimated. Plane mergence is performed at last to append the missed cuboid faces.

Details of all four modules are explained respectively in the following subsections.

### 2.1. Preprocessing

#### 2.1.1. Removal of Outliers

**Figure 2:**
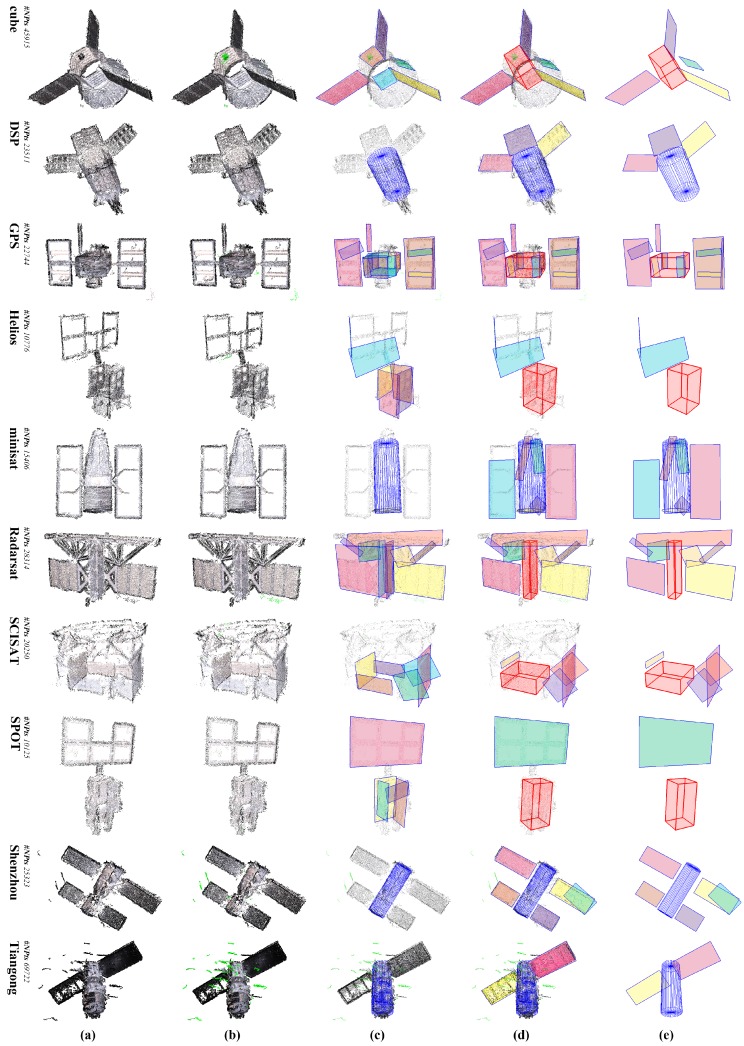
Detection results of the reconstructed point cloud data. From left to right: (**a**) the origin input point clouds; (**b**) results of outliers removal, where the identified outliers are colored in green; (**c**) results of cylinder detection or patch detection; the cylinders are rendered in blue, and patches are rendered in different colors; (**d**) final results; the detected cuboids are rendered as red boxes; (**e**) a clear view of the detected components. The number of points (#NPts) is noted above model names.

Point cloud data generated by laser scanning or image-based reconstruction usually contain outliers that drift far away from the object. Statistical analysis with the k nearest neighbor (KNN) can effectively classify outliers that are randomly distributed. However, outliers could be also structured where outliers are in the form of high-density continuous clusters separating from the object points, especially for point cloud data obtained by image-based reconstruction, as shown in Figure 2. To remove such outliers, the input point clouds are clustered into groups by region growing in 3D space, and the largest group with the most points is selected as the point set of the object, while other groups are regarded as outliers. Results of outliers’ removal are displayed in the second column of Figure 2b, where the removed outliers are colored in green.

#### 2.1.2. Estimation of Dimensional Unit ε

To handle the scale variation in different point cloud data, an adaptive dimensional unit ε is estimated for usage in the subsequent detection procedures. Two reference lengths $l_{D}$ and $l_{R}$ are used to estimate ε. $l_{D}$ is the minimum length of the edges of the minimum enclosing box of the 3D point data P. $l_{R}$ is a length that measures the surface roughness of P. For each point p∈P, a local plane $\pi_{p}$ can be estimated by least square fitting with its *k* nearest neighbors $N_{k}\left(p\right)$. $l_{R}$ is then defined as:

(1) $$l_{R}=\frac{1}{\left|P\right|}\sum_{p\in P}a\mu_{p}+b\sigma_{p},$$

where $\left|P\right|$ is the size of P, $\mu_{p}$ and $\sigma_{p}$ are the mean and standard deviation of the distances from $N_{k}\left(p\right)$ to $\pi_{p}$ and *a* and *b* are two coefficients, which are set as a=1 and b=3 in practice. Then, the dimensional unit ε is defined as:

(2) $$\varepsilon =\min(\beta l_{D},\max\left(\alpha l_{D},l_{R}\right)),0<\alpha <\beta <1,$$

where α and β are two small factors with α=1 and β=0.03 respectively in this paper. The definition of dimensional unit ε considers both point cloud dimensions and surface roughness. Dimension length $l_{D}$ enables ε to handle the scale variation, while roughness length $l_{R}$ makes ε robust to point position noise. Meanwhile, the upper and lower bounds determined by α and β achieve a trade-off between precision and computational consumption.

### 2.2. Detection of Cylinders

Multiple cylinder proposals are initially generated in a RANSAC paradigm, and the desired cylinder primitives are then detected in iterations of energy-based multi-model fitting [26] and cylinder parameter estimation [31]. Surface points of the detected cylinders are finally verified by distance proximity and orientation proximity, and points that do not belong to cylinders will be taken as the input of the subsequent path detection.

#### 2.2.1. Generation of Initial Cylinders

To estimate a cylinder, only two points with the normal are needed. Given cylinder surface points *p* at $c_{p}$ with normal $n_{p}$ and points *q* at $c_{q}$ with normal $n_{q}$, the axis of the cylinder can be found as the common perpendicular line between $n_{p}$ and $n_{q}$ with direction $n_{p}\times n_{q}$. Meanwhile, the radius of the cylinder is estimated as the average distance of $c_{p}$ and $c_{q}$ to the axis. The initial cylinders are generated with random sampled point pairs by RANSAC. Notice that obviously improper cylinder estimations are discarded, such as the cylinder estimated in the cases:

- the distances from $c_{p}$ and $c_{q}$ to the axis are inconsistent;
- the center of the cylinder is out of the minimum enclosing box of the point clouds;
- the radius of the cylinder exceeds the minimum dimension of the minimum enclosing box;
- the inlier percentage is less than preset threshold $T_{cyl}$.

#### 2.2.2. Energy-Based Multi-Cylinder Fitting

Given a point *p* in point cloud P, $f_{p}\in L$ is the label assigned to *p*, i.e., *p* is classified as a point of the cylinder associated with label $f_{p}$. Then, multiple cylinders are detected by minimizing energy E(L) of labeling $L=\{f_{p}|p\in P\}$ for all points as:

(3) $$E\left(L\right)=\sum_{p\in P}D_{p}\left(f_{p}\right)+\sum_{(p,q)\in N}V_{pq}\left(f_{p},f_{q}\right)+\eta \left|L_{L}\right|.$$

Energy E(L) consists of three terms, i.e., data term $D_{p}\left(f_{p}\right)$, smooth term $V_{pq}\left(f_{p},f_{q}\right)$ and label cost $\eta |L_{L}|$. Their definitions are as follows:

- Data term $D_{p}\left(f_{p}\right)$ is a geometric error energy, which measures the disagreement between *p* and the corresponding cylinder model $\mathrm{Cyl}(f_{p})$. It is defined as the sum of distance proximity and orientation proximity, i.e.,
  (4) $$D_{p}\left(f_{p}\right)=∥p-\mathrm{Cyl}\left(f_{p}\right)∥=\frac{D_{dist}\left(f_{p},\mathrm{Cyl}\left(f_{p}\right)\right)}{\varepsilon }+\frac{D_{ang}\left(f_{p},\mathrm{Cyl}\left(f_{p}\right)\right)}{d_{\Delta }},$$
  where $D_{dist}$ is the distance variance of *p* to the surface of $\mathrm{Cyl}(f_{p})$, $D_{ang}$ is the angle variance of the normal of *p*. $D_{dist}$ and $D_{ang}$ are separately discretized with resolution ε and $d_{\Delta }$, and $d_{\Delta }$ is set to 15° in our experiments.
- Smooth term $V_{pq}\left(f_{p},f_{q}\right)$ is a discontinuity preserving smoothness error energy, which constrains the labeling consistency among the neighboring points. In our scheme, the neighborhood system N employs the KNN. $V_{pq}\left(f_{p},f_{q}\right)$ is described by the Potts model [29] as:
  (5) $$V_{pq}\left(f_{p},f_{q}\right)=\lambda \omega_{pq}\delta \left(f_{p}\neq f_{q}\right),$$
  where λ is a weight coefficient and δ(·) is an indicator, which takes one if its argument is true, and otherwise zero. $\omega_{pq}$ is the coefficient to penalize the discontinuity of neighboring points, with the definition as:
  (6) $$\omega_{pq}=\exp\left(-\frac{{\left(∥p-q∥/\varepsilon \right)}^{2}}{\gamma^{2}}\right),$$
  where γ is a constant coefficient.
- Label cost $\eta |L_{L}|$ is the label energy to prevent over-fitting, where $L_{L}$ is the set of distinct labels assigned to data points by labeling L, $|L_{L}|$ is the cardinality of $L_{L}$ and η is a coefficient.

To handle the outliers, a special outlier model is introduced with label notation *∅*. Points assigned this label are considered as outliers, and the data term for *∅* is a constant $e_{\emptyset }$, i.e., $∥p-\emptyset ∥=e_{\emptyset }$. Then, the energy function for multi-cylinder detection can be expressed as:

(7) $$E\left(L\right)=\sum_{p\in P}∥p-\mathrm{Cyl}\left(f_{p}\right)∥+\lambda \sum_{(p,q)\in N}\omega_{pq}\delta \left(f_{p}\neq f_{q}\right)+\eta \left|L_{L}\right|.$$

Minimization of this energy function can be effectively solved by the extended graph-based α-expansion method [28].

#### 2.2.3. Iteration of Model Fitting and Estimation

The energy-based multi-model fitting will deliver a labeling result, where points in P are classified into different cylinders or outliers. The cylinders are then refined via least square fitting (LSF) [31] with their support (inliers). The error function of LSF is the total squared distance error of the input point data to the surface of the cylinder, and parameter estimation is performed by searching a large number of directions and finding the optimal radius and location. In the model selection step in Figure 1, cylinders with a low inlier percentage are discarded, and those close to each other are merged. We update the initial models of energy-based model fitting with these refined cylinders and iteratively perform the fitting and estimation steps to get a better set of cylinders. As the iteration could rapidly converge to a stable result within just a few times, the max iteration time is set to three in our experiments.

### 2.3. Detection of Planar Patches

#### 2.3.1. Plane Detection

In our detection scheme, planes in 3D space are iteratively detected by the 3D Hough transform method. A 3D plane Π can be formulated as $s_{x}x+s_{y}y+s_{z}z+s_{d}=0$ in Cartesian coordinates. To uniquely define the plane, one parameter among $s_{x}$, $s_{y}$ and $s_{z}$ is often fixed in advance, e.g., set $s_{x}$ to one. In this case, planes in specific orientations will result in rather small (perpendicular to the *X*-axis) or large (parallel to the *X*-axis) values of $s_{y}$ and/or $s_{z}$. Namely, both large range and small accuracy are required for $s_{y}$ and $s_{z}$, which is obviously memory and time consuming. To handle this problem, our scheme uses three separate Hough transformations in one detection. For each Hough transformation, one parameter among $s_{x}$, $s_{y}$ and $s_{z}$ is fixed to 1, and the other two are discretized in the range [−1,1]. The desired plane is finally determined by the most voted point among all three Hough transformations. To accelerate the computation, these three Hough transformations are performed in parallel. Meanwhile, the voting is constrained with the normals, i.e., only planes nearly perpendicular to the normal of the space point are voted for, and this also accelerates the computation greatly. In this paper, $s_{x}$, $s_{y}$ and $s_{z}$ are discretized in resolution 0.01, and $s_{d}$ is discretized with the dimensional unit ε estimated in the preprocessing stage.

Given a detected plane, its surface points are then verified by distance proximity and orientation proximity. However, these points do not necessarily belong to the same plane. It may consist of multiple coplanar planes and the parts of the component surfaces that intersect this plane. To distinguish these points, a distance-proximity-based region-growing approach is utilized. The largest group with most points is kept, while the other points are put into the next iteration of plane detection.

#### 2.3.2. MBR Extraction

Since the planes are actually bounded planar patches rather than infinitely extended ones, the representation of the detected plane primitive is further improved to a patch by finding its minimum bounding rectangle. Notice that the plane for minimum bounding rectangle calculation is refined by least square fitting, and all these surface points are projected to the fitted plane. There may be outlier points making wrong bounding rectangle, and such outliers usually locate at the bound of the wrong rectangle and could result in a significant increase of the area of the bounding rectangle. The outlier bounding points are detected by local density and removed during the MBR extraction. The specific details of the MBR extraction are explained in Algorithm 1.

**Algorithm 1:** Robust extraction of minimum bounding rectangle.
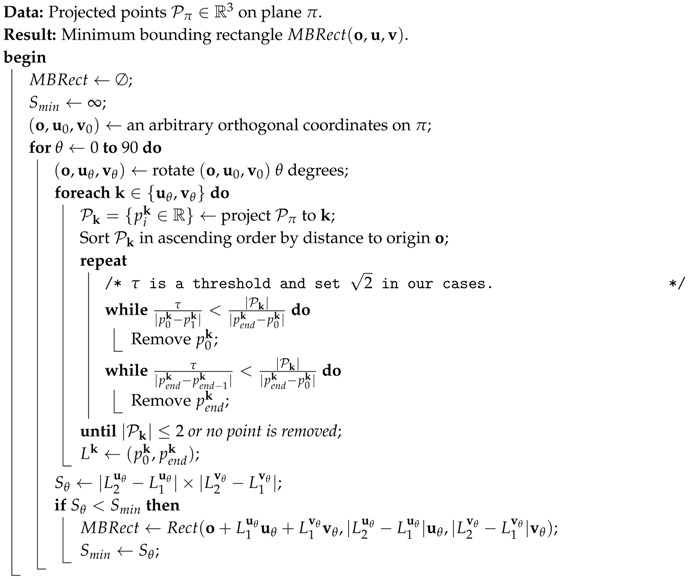


Multiple patches are detected by performing plane detection and MBR extraction iteratively, until no point remains or the inlier percentage of the current detected patch is lower than a preset threshold $T_{ple}$. Note that in our proposed scheme, the circle patch is considered as a special rectangle patch in plane detection and distinguished by calculating its length-to-width ratio and point distribution in its inscribed circle.

### 2.4. Detection of Cuboids

#### 2.4.1. Geometry Relation Criteria

Faces of the cuboid main bodies, if they exist, may also be detected as patches in the plane detection stage. Therefore, a geometry relationship inferring approach is proposed to distinguish these patches. As for a cuboid, there are two kinds of pair-wise geometry relations among its six faces, the opposite faces and the adjacent faces. Our scheme exploits the following criteria to identify the relations.

- Criterion for two opposite faces: (i) their plane normals are parallel, and their edges are respectively parallel, as well; (ii) after projecting each patch to the other and calculating the ratio of intersection over union (IoU), the average IoU should be greater than a preset threshold $T_{IoU}$.
- Criterion for two adjacent faces: (i) their plane normals are perpendicular, and their edges are respectively parallel or perpendicular; (ii) some edge of one patch is adjacent to that of the other with similar lengths.

**Figure 3:**
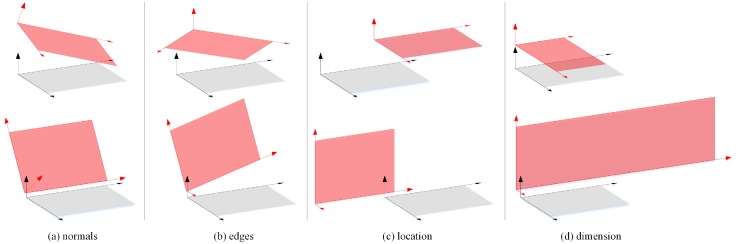
Criteria for opposite faces (the top row) and adjacent faces (the bottom row). The gray patch is a reference patch, and the red ones are patches that do not satisfy the (**a**) normal direction; (**b**) edge directions; (**c**) relative location or (**d**) dimension requirements.

For criteria of both opposite and adjacent faces, Condition (i) constrains the orientation relations so that patch pairs with improper relative normal directions (Figure 3a) or edge directions (Figure 3b) can be filtered; while Condition (ii) constrains both the relative location and dimension so that patch pairs with a dramatic position deviation (Figure 3c) or unmatched dimensions (Figure 3d) can be filtered. It should be noted that the parallel and perpendicular relations are determined by a given maximal angle error $T_{\theta }$ in this paper.

Through these geometry relation criteria, we can generate multiple cuboid face patch groups, among which each pair of patches satisfies the opposite or adjacent criterion. Meanwhile, these groups must agree with the cuboid configure, i.e., one cuboid face patch could have only one opposite patch and four adjacent patches at most, and the adjacent patches should be located at different directions. Face patch groups containing more than three faces or at least one pair of opposite faces is used to estimate the cuboids.

#### 2.4.2. Orientation Estimation

A face patch group of a cuboid *C* can be defined as $S_{c}\left\{P_{i}\right\}$. A face patch $P_{i}$ is defined as a combination of vectors, i.e., $P_{i}=\left\{o_{i},n_{i},u_{i},v_{i}\right\}$, where $n_{i}$ is the normalized normal, $o_{i}$ is one reference vertex of $P_{i}$ and $u_{i}$ and $v_{i}$ are the corresponding edges with lengths $|u_{i}|$ and $|v_{i}|$. All these elements are 3×1 vectors. The orientation of cuboid *C* is first estimated with $S_{c}$. For each face direction $k\in \{n_{x},n_{y},n_{z}\}$ of cuboid *C*, where $n_{x}$, $n_{y}$ and $n_{z}$ are the 3×1 normals of three orthogonal faces of *C*, $S_{c}$ can be divided into two groups, i.e., perpendicular patches $S_{⊥}^{k}\left\{P_{i}|P_{i}\in S_{c},P_{i}⊥k\right\}$ and parallel patches $S_{∥}^{k}\left\{P_{i}|P_{i}\in S_{c},P_{i}∥k\right\}$. Then, k can be estimated as:

(8) $$\hat{k}=\left\{\begin{array}{cc} \frac{\sum_{P_{i}\in S_{⊥}^{k}}W\left(n_{i}\right)n_{i}}{\sum_{P_{i}\in S_{⊥}^{k}}W(n_{i})} & S_{⊥}^{k}\neq \emptyset ,\\ \frac{\sum_{P_{i}\in S_{∥}^{k}}W\left(u_{i}\right)\delta \left(k⊥u_{i}\right)\frac{u_{i}}{|u_{i}|}+W\left(v_{i}\right)\delta \left(k⊥v_{i}\right)\frac{v_{i}}{|v_{i}|}}{\sum_{P_{i}\in S_{∥}^{k}}W\left(u_{i}\right)\delta \left(k⊥u_{i}\right)+W\left(v_{i}\right)\delta \left(k⊥v_{i}\right)} & \mathrm{otherwise}. \end{array}\right.$$

In Equation (8), δ(p⊥q) is an indicator, and it takes one if p and q are parallel, and otherwise zero. $W(n_{i})$ is a coefficient defined as the reciprocal of fitting error of the plane. $W(u_{i})$ and $W(v_{i})$ are confidence coefficients that indicate the uniformity of the points distributing along $u_{i}$ and $v_{i}$. Given an edge q∈{u,v} of a patch *P*, points $P_{P}$ belonging to *P* are first projected to q, and then, the distribution histogram of the projected points can be computed within $N_{Bin}$ ($N_{Bin}=10$ in our experiments) bins. Thus, W(q) can be defined as:

(9) $$W\left(q\right)=\frac{\sum_{i=1}^{N_{Bin}}-\frac{n_{i}}{|P_{P}|}\ln\frac{n_{i}}{|P_{P}|}}{\ln N_{Bin}},$$

where $n_{i}$ is the number of points falling in the *i*-th bin and $|P_{P}|$ is the number of points in $P_{P}$. The numerator represents the distribution entropy and can reach the maximum value (i.e., the denominator) when the histogram is uniformly distributed.

During orientation estimation of cuboid *C*, patch normals are preferred to be utilized as they are generally more accurate and credible than edges. In addition, to guarantee the orthogonality of the estimated face normals, the diagonal matrix of singular value decomposition (SVD) of the direction matrix $\left[\hat{n_{x}},\hat{n_{y}},\hat{n_{z}}\right]$ is forced to be an identity.

#### 2.4.3. Dimension Estimation

The dimensions of cuboid *C* are estimated after orientation estimation. For each direction $k\in \{n_{x},n_{y},n_{z}\}$, vertexes of all the patches in $S_{c}$ are projected to k, and the projected points would distribute in two clusters, $P_{k}^{1}\left\{p_{i}\in R\right\}$ and $P_{k}^{2}\left\{p_{i}\in R\right\}$. $P_{k}^{1}$ represents that group that is closer to the origin o, and $P_{k}^{2}$ represents the farther one. Therefore, the dimension along k is defined as $l_{k}=abs\left(p_{k}^{2}-p_{k}^{1}\right)$, and $p_{k}^{n}$ is the clustering center of $P_{k}^{n}$, which can be computed as:

(10) $$p_{k}^{n}=\left\{\begin{array}{cc} \mathrm{mean}(P_{⊥k}^{n}) & P_{⊥k}^{n}\neq \emptyset ,\\ \mathrm{median}(P_{k}^{n}) & \mathrm{otherwise}. \end{array}\right.,\;n=1,2.$$

$P_{⊥k}^{n}\subset P_{k}^{n}$ consists of project points of patch $P_{⊥k}^{n}\in S_{⊥}^{k}$. The cuboid is finally represented as $C=(o+p_{x}^{1}n_{x}+p_{y}^{1}n_{y}+p_{z}^{1}n_{z},l_{x}n_{x},l_{y}n_{y},l_{z}n_{z})$.

#### 2.4.4. Plane Mergence

**Figure 4:**
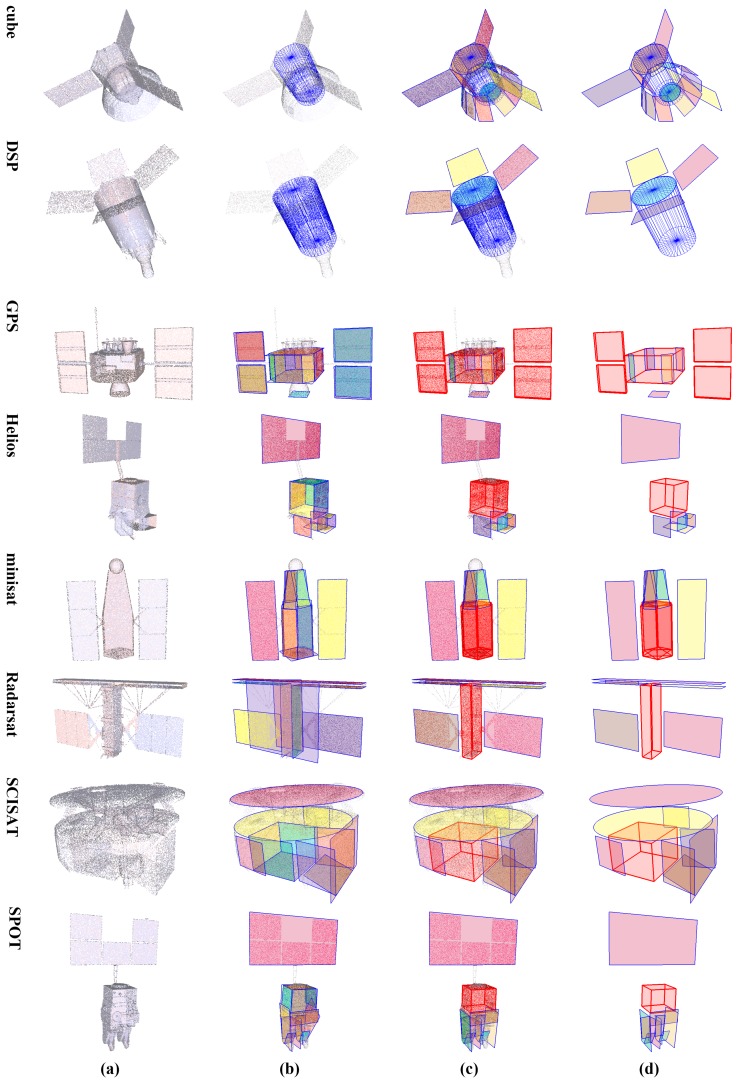
Detection results of the synthesized point cloud data *50K_00U_00D*. From left to right: (**a**) the origin input point clouds; (**b**) results of cylinder detection or patch detection after detection of cylinders (the cylinders are rendered in blue, and patches are rendered in different colors.); (**c**) final results (the detected cuboids are rendered as red boxes.); (**d**) a clear view of the detected components.

The cuboid face patches may be incorrectly detected as a larger patch along with other patches. For example, the front and back faces of the cuboid main body in spacecraft Radarsat are detected as bigger patches along with the points of its antenna, as shown in Figure 4c. To correctly pick out the cuboid face patches, patches that are close to faces of cuboid *C* will be partly merged into *C*, and the MBRs of the remaining parts of such patches will be updated.

## 3. Experiments

### 3.1. Data Collection and Parameter Configure

Both synthesized spacecraft point cloud data and those recovered by image-based reconstruction are tested in our experiments.

**Figure 5:**
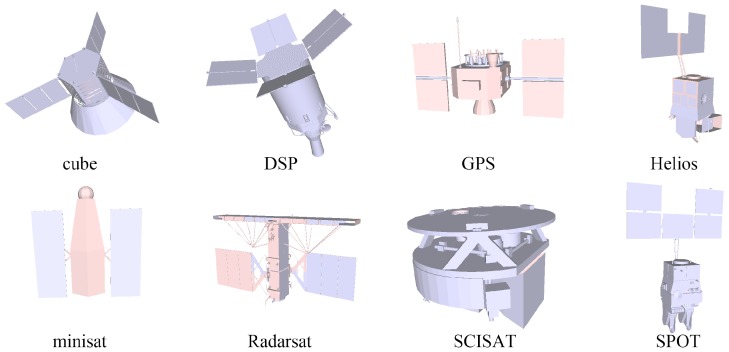
Origin CAD mesh models of 8 spacecraft. Synthesized point clouds are generated by sampling surfaces of these models.

- Synthesized point clouds: The synthesized point clouds are generated by uniformly sampling the surfaces of 8 spacecraft computer-aided design (CAD) models in the BUAA space object image dataset (BUAA-SID) [4]. Origin CAD mesh models of the 8 spacecraft are shown in Figure 5. To test our scheme and evaluate its robustness, synthesized data with different point distribution densities, position noise and direction noise are generated. To add position noise, a deviation of $k_{u}n$ along a random direction is added to every point. *n* is a standard Gaussian noise, and $k_{u}$ is a distance deviation coefficient, which takes $1\times (0.01\times l_{D})$, $2\times (0.01\times l_{D})$ or $4\times (0.01\times l_{D})$ ($l_{D}$ is the dimension length described in Section 2.1). To add direction noise, a rotation of a random direction with angle $k_{d}n$ is applied to the normal of every point. $k_{d}$ is a direction deviation coefficient, which takes 5°, 10° or 15°. We use {01U, 02U, 04U} and {05D, 10D, 15D} to denote different levels of position and direction noise, respectively.
  Synthesized point cloud of each spacecraft containing 50Knoise-free points are used for basic testing. For robustness analysis on density, point clouds with 20K, 10K and 05K noise-free samples are used. For robustness analysis on position noise, point clouds with 50K samples and distance noise levels of 01U, 02U and 04U are used. For robustness analysis on direction noise, point clouds with 50K samples and direction noise levels of 05D, 10D and 15D are used. In addition, point clouds with 20K, 10K and 05K samples, distance noise level of 04U and direction noise level of 15D are tested for integrated analysis. For clarity, each synthesized point cloud is referred to as **K_**U_**D, where **K, **U and **D denote the sample number, distance noise level and direction noise level, respectively. For example, a point cloud with 50K samples, distance noise level of 04U and direction noise level of 15D is referred to as *50K_04U_15D*.
- Reconstructed point clouds: the reconstructed point cloud data are recovered from simulated images sequences of the 8 spacecraft models via the image-based reconstruction method [14]. Besides, reconstruction results of real scaled models of spacecraft Shenzhou and Tiangong [14] are also included reconstructed point cloud data. Reconstructed point clouds of these 10 models are shown in the first column in Figure 2.

Several preset thresholds are used in our scheme, including inlier rate threshold for patch detection $T_{ple}$ and cylinder detection $T_{cyl}$, thresholds used for cuboid detection in geometry relation criteria $T_{IoU}$ and $T_{\theta }$, distance proximity threshold $T_{dist}$ and orientation proximity threshold $T_{ang}$ used for surface point verification of both planes and cylinders. Coefficients used in the energy function for cylinder fitting are adaptively set as $e_{\emptyset }=\lambda =\gamma =2\times T_{dist}$, $\eta =T_{cyl}\times \left|P\right|$. Definitions of these coefficients can be found in Section 2.2. Other fixed coefficients not mentioned here are given where they appear in Section 2. Since these parameters are robust, we used the same threshold configures for most experiments as listed in Table 1. An exception $T_{ang}$ takes 15° for synthesized data **K_**U_00D and **K_**U_05D and 30° to handle heavier direction noise for **K_**U_15D and the reconstructed data.

### 3.2. Results on Synthesized Point Clouds

Spacecraft component detection results of synthesized point cloud data *50K_00U_00D* are shown in Figure 4. It can be seen that the major components of these spacecraft are precisely detected by our scheme. Since arbitrary prisms (a cuboid is a special quadrangular prism) and cones are currently not covered in our scheme, they will be detected as other proximate geometric primitives. The hexagonal prism in spacecraft cube is detected as a cylinder, while the hexagonal prism in spacecraft minisat is detected as three cuboids, which are supported by only a pair of opposite faces. Besides, the cone in spacecraft cube is detected as patches. Such results may not be accurate, but still quite appropriate. Moreover, although the front and back faces of the cuboid main body in spacecraft Radarsat are detected as bigger patches along with some points of its antenna, these cuboid faces are effectively picked out in the patch mergence step, and the overall result is quite fine. The effectiveness of our detection scheme is thus demonstrated.

### 3.3. Robustness Analysis

**Figure 6:**
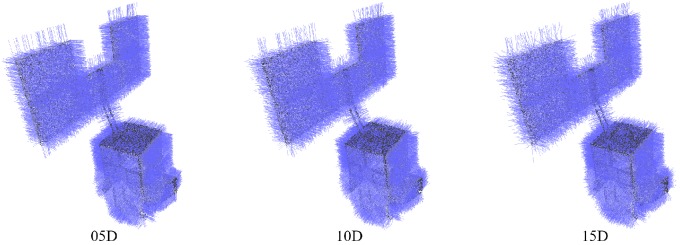
Point clouds with normals rendered. Point clouds of spacecraft Helios with different direction noise levels.

To further evaluate the robustness of our scheme, we perform experiments on synthesized point cloud data with different factors, including point distribution density, position noise and direction noise. Note that the position noise can be directly seen from the point clouds, while the direction noise can be observed only when normals are rendered, as shown in Figure 6.

**Figure 7:**
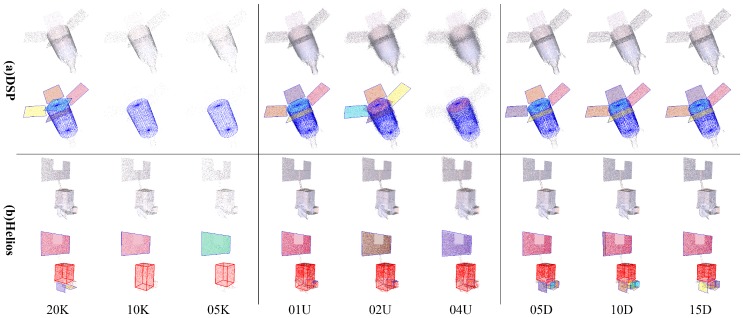
Results of robustness analysis with a single factor. The first grid row is the detection results of spacecraft (**a**) DSP, and the second grid row is the results of (**b**) Helios. From left to right, the grid columns are point clouds with different sample numbers (**K_00U_00D), different distance noises (50K_**U_00D) and different direction noises (50K_00U_**D). The top row in each grid displays the origin input point clouds, and the bottom row displays the final results. The cylinders are rendered in blue; patches are rendered in different colors; and cuboids are rendered as red boxes.

Results of robustness analysis with a single factor are illustrated in Figure 7. It should be noticed that only selected results of spacecraft DSP and Helios are displayed in Figure 7, and the results of all 8 spacecraft with intermediate detection results can be found in our Supplementary Material.

The first grid column in Figure 7 demonstrates the result of point cloud data with different sample numbers. The inlier rate would get lower as the density gets sparser, thus leading to missed detection of small components; for example, the solar panels of spacecraft DSP and below the structure of Helios in point clouds *10K_00U_00D*, *05K_00U_00D* and *50K_04U_00D*. Nevertheless, the major components are always effectively detected. The second and third grid columns in Figure 7 are the results of points cloud data with different distance noise and direction noise, where only solar panels of spacecraft DSP in point clouds *50K_04U_00D* are missed. In general, the proposed scheme copes with the density variation and has fine robustness to noise.

**Figure 8:**
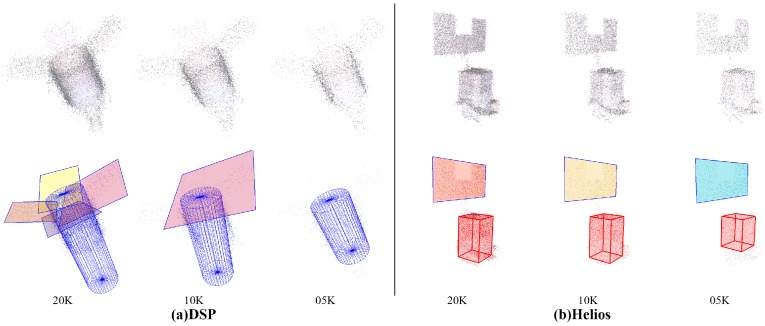
Results of robustness analysis with integrated factors. The left grid column is the detection results of spacecraft (**a**) DSP, and the right grid column is the results of (**b**) Helios. The top row in each grid displays the origin input point clouds, and the bottom row displays the final results. From left to right in each grid are point clouds with different sample numbers (**K_04U_15D). The cylinders are rendered in blue; cuboids are rendered as red boxes; and patches are rendered in different colors.

An integrated analysis with multiple factors is further conducted, in which the proposed scheme is tested on point clouds with a noise level of 4U and 15D (**K_04U_15D). The results in Figure 8 show that the major components are effectively detected and the overall results are still quite fine. As stated above, our proposed scheme can robustly achieve spacecraft component detection.

### 3.4. Results on Reconstructed Point Clouds

Reconstructed point clouds are more challenging, because: (a) these point clouds contain severe noise and numerous outliers; (b) many parts of the surface failed to be recovered due to their lack of textures; and (c) the points are not uniformly distributed as synthesized data either. However, as shown in Figure 2, detection results of these reconstructed point clouds are gratifying. Components in the spacecraft models are effectively detected. Two faces of hexagonal prisms in a cube are detected, from which a cuboid is generated. The hexagonal prism in minisat is detected as a whole cylinder. Such situations are quite acceptable since we focus on detecting geometric primitives like plane, cuboid and cylinder. In addition, similar to Figure 4, the front face of the main body in Radarsat is again detected as a whole patch along with its left solar panel, but it is correctly handled by patch mergence.

**Figure 9:**
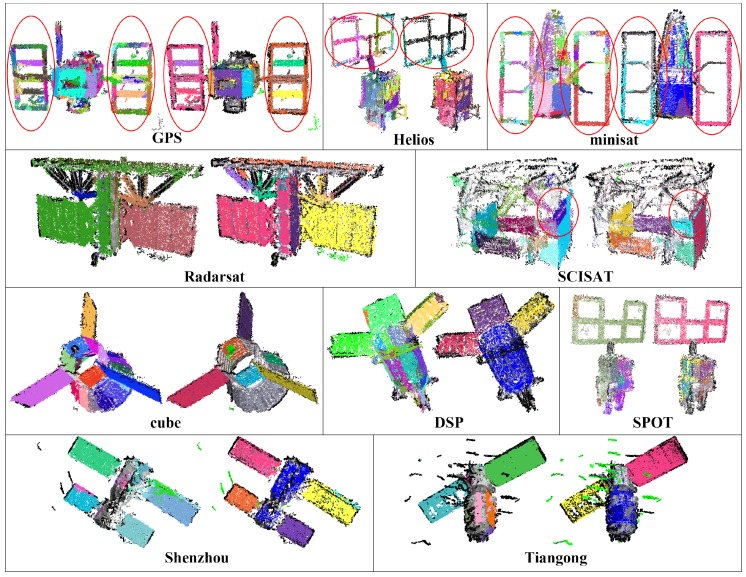
Comparison of plane detection results of [25] (the left ones) and our scheme (the right ones). In [25], no further bounded patch model is generated, and points are rendered in different colors to indicate different planes. For convenience of comparison with [25], the results of ours are rendered in the same pattern. The main differences are marked with red circles.

Figure 9 shows the plane detection results of [25] and our scheme on the reconstructed point cloud data. In [25], the 3D points are stored as an octree, and planes are detected by RANSAC. Note that [25] focuses only on the detection of planes; outliers are not specially concerned, and no further bounded patch model is generated either. As shown in Figure 9, the result points of [25] (the left ones) are rendered with different colors according to their verification results. For convenient comparison with [25], results of our scheme just after the patch detection step are also given in Figure 9 (the right ones), which are rendered with the same pattern as the results of [25]. We can see that the noise of these point cloud data causes many undesired planes in the results of [25], such as the solar panel areas of spacecraft GPS, Helios and minisat. Moreover, the division nature of octree in [25] could also lead to additional planes; for example, the blue plane of metric SCISAT as emphasized in Figure 9; while the planes generated by our scheme are more compact and clean. The reason mainly lies in the employment of adaptive dimensional unit ε, which makes our scheme robust to both scale variation and noise.

**Figure 10:**
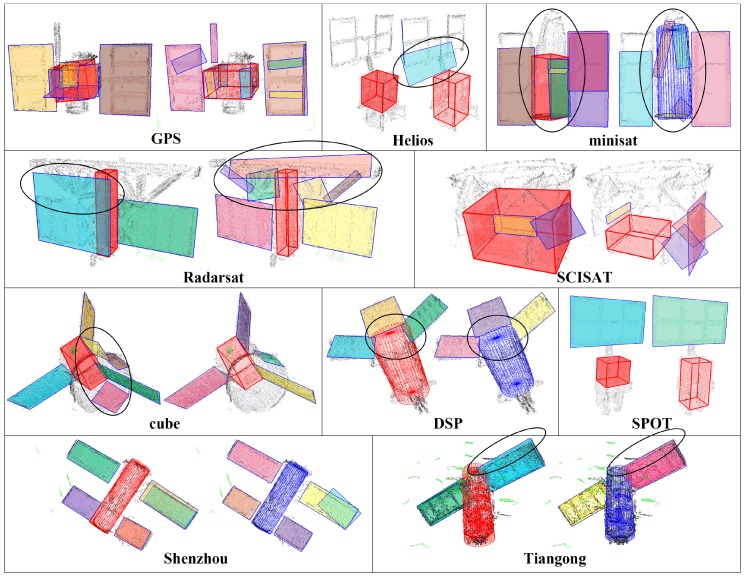
Comparison of component detection results of [30] (the left ones) and our scheme (the right ones). The identified outliers are colored in green; patches are rendered in different colors; the detected cuboids are rendered as red boxes; and the detected cylinders are rendered as red or blue wire-frame.

Figure 10 gives the comparison of the component detection results of [30] (the left ones) and our scheme (the right ones). Since this paper is a further improvement of [30], the approach in [30] is similar to that of this paper, except that: (a) the cylinder is detected with a novel axial-symmetry-based cylinder detection method lastly, rather than at beginning; (b) the MBR estimation approach is not improved; and (c) the patch mergence step is not introduced either. Through the comparison of Figure 10, we can see that: (a) the cylinders are more precisely detected due to utilization of more robust cylinder detection, for instance the cylinder main body of DSP; (b) contours of the detected patches are also better fitted due to improvement on MBR estimation, for instance the solar panels of GPS and Tiangong; and (c) the cuboids are better extracted due to the patches mergence steps, for instance the cuboid main body of Radarsat.

To quantitatively evaluate the accuracy of the generated models, the evaluation in [32,33] is utilized, which was originally designed for accuracy evaluation of multi-view reconstruction. Let the reconstructed point clouds be the reference point clouds R; we first convert the generated geometric model into point clouds M by uniform sampling, where the sample number is equal to the point number of R. Then, the precision measurement P(d) for distance threshold *d* is defined as:

(11) $$P\left(d\right)=\frac{100}{\left|M\right|}\sum_{m\in M}\left[e_{m\to R}<d\right]$$

where $e_{m\to R}=\min_{r\in R}\left|m-r\right|$ is the distance of point m∈M to R, and [·] is the Iverson bracket. Similarly, the completeness measurement C(d) for distance threshold *d* is defined as:

(12) $$C\left(d\right)=\frac{100}{\left|R\right|}\sum_{r\in R}\left[e_{r\to M}<d\right]$$

where $e_{r\to M}=\min_{m\in M}\left|r-m\right|$ is the distance of point r∈R to M. In addition, the F-score F(d) is computed to combine the precision and completeness measurements, which is defined as the harmonic mean of P(d) and C(d), i.e.,

(13) $$F\left(d\right)=\frac{2P(d)C(d)}{P(d)+C(d)}.$$

In this paper, we use the measurements P(d), C(d) and F(d) to evaluate the accuracy of the generated models, and the distance threshold *d* is set to d=2ε. C(d) is determined by the precision (location, orientation and dimension) of the generated model and detection capability. Poor and unsuccessful detections would lead to large $e_{r\to R}$ and low C(d); while P(d) is mainly determined by the detection precision and influenced by the completeness of the input point cloud. Missed parts of the input point cloud, such as hollows and sparse areas, would lead to large $e_{m\to R}$, thus resulting in low P(d).

The evaluation of detection results of both [30] and ours is listed in Table 2. Our scheme outperforms [30] on both measurements P(d) and C(d) for most spacecrafts, except cube, Helios, minisat and Radarsat. As for spacecraft minisat, the hexagonal prism is detected as planes and cuboid by [30], while our scheme treats it as planes and cylinder. Since cuboid fits the hexagonal prism better than cylinder, method in [30] obtains higher scores on both C(d) and F(d). As for Helios and Radarsat, additional planes, where the point clouds are sparse, are only detected by our scheme, and it causes the lower P(d). The additional detected planes are however valid and contribute to higher completeness of measurements C(d) and F(d), which means that our detection results are more complete. Similarly, method in [30] detects two more planes on cube than our scheme and thus achieves a higher C(d). However, these two planes are not sparse; therefore, the precision measurement P(d) of [30] on cube is not lower than ours.

In summary, our proposed scheme outperforms [30] on both regularization and accuracy in most cases, which demonstrates the effectiveness and accuracy of our scheme.

## 4. Conclusions

In this paper, we have proposed a novel component detection scheme to automatically detect the basic components of spacecraft. The basic components, including solar panels, cuboid and cylinder modules, are treated as geometric primitives such as planar patches, cuboids or cylinders. We revised the detection procedure of previous work [30], utilized a more robust cylinder detection, improved MBR estimation, and introduced a patch mergence step. The performance of the proposed scheme was evaluated on synthesized spacecraft point cloud data and more challenging reconstructed point cloud data. Experimental results demonstrate the effectiveness, robustness and accuracy of our spacecraft component detection scheme. In future work, cones, arbitrary prisms and general polygons can be further considered in spacecraft component detection to make it more comprehensive.

## Figures and Tables

**Table 1:** Thresholds used in our detection scheme. IoU, intersection over union.

Notation	Definition	Value
$T_{ple}$	Inlier rate threshold for planes in patch detection.	0.05
$T_{cyl}$	Inlier rate threshold for cylinders in cylinder detection.	0.1
$T_{IoU}$	IoU threshold for opposite faces in patch-wise geometry relation criteria.	0.6
$T_{\theta }$	Angle deviation for parallel and perpendicular patches.	15°
$T_{dist}$	Distance proximity threshold for surface point verification.	2×ε
$T_{ang}$	Orientation proximity threshold for surface point verification.	15°∼30°

**Table 2:** Accuracy evaluation of the generated geometric models.

Objects	Method in [30]			Our Scheme		
	P(d=2ε)	C(d=2ε)	F(d=2ε)	P(d=2ε)	C(d=2ε)	F(d=2ε)
cube	**72.0**	**71.5**	**71.7**	70.5	64.2	67.2
DSP	80.9	73.6	77.1	**89.8**	**77.0**	**82.9**
GPS	46.3	50.1	48.2	**64.4**	**71.6**	**67.8**
Helios	**61.8**	36.5	45.9	48.7	**50.7**	**49.7**
minisat	**33.6**	**48.1**	**39.6**	28.6	39.0	33.0
Radarsat	**80.0**	60.9	69.1	77.3	**71.3**	**74.2**
SCISAT	27.8	42.3	33.6	**51.0**	**42.6**	**46.4**
SPOT	37.1	37.9	37.5	**39.5**	**41.5**	**40.5**
Shenzhou	81.3	80.3	80.8	**87.3**	**85.7**	**86.5**
Tiangong	86.0	84.2	85.1	**95.4**	**86.5**	**90.7**

## Supplementary Materials

Detailed component detection results of robustness analysis are available in supplementary: http://www.mdpi.com/1424-8220/18/4/933/s1, including results for different point distribution density (20K, 10K and 05K), position noise (01U, 02U and 04U) and direction noise (05D, 10D and 15D) of all the 8 spacecraft (cube, DSP, GPS, Helios, minisat, Radarsat, SCISAT and SPOT), along with the intermediate detection results.
