# Supplementary material for: Robust Spacecraft Component Detection in Point Clouds
Source: Sensors (Basel). 2018 Mar 21;18(4):933. doi: 10.3390/s18040933 (PMC5949025; doi:10.3390/s18040933)
Supplement: Supplementary file 1 [file sensors-18-00933-s001.pdf]

Supplementary Materials: Robust Spacecraft Component Detection in Point Clouds

Quanmao Wei [1,2], Zhiguo Jiang [1,2*] and Haopeng Zhang [1,2*]

This is the supplementary for article *Robust Spacecraft Component Detection in Point Clouds* submitted to *Sensors*.

This supplementary contains component detection results of robustness analysis of the proposed detection scheme, details of which can be referred to the text of the article. Results for different point distribution density (**20K**, **10K** and **05K**), position noise (**01U**, **02U** and **04U**) and direction noise (**05D**, **10D** and **15D**) of all the 8 spacecraft (**cube**, **DSP**, **GPS**, **Helios**, **minisat**, **Radarsat**, **SCISAT** and **SPOT**) with intermediate detection results are displayed in this supplementary. Definitions of notations for distribution density and noise level, e.g., 50K, 04U and 15D, can be referred to text of the article.

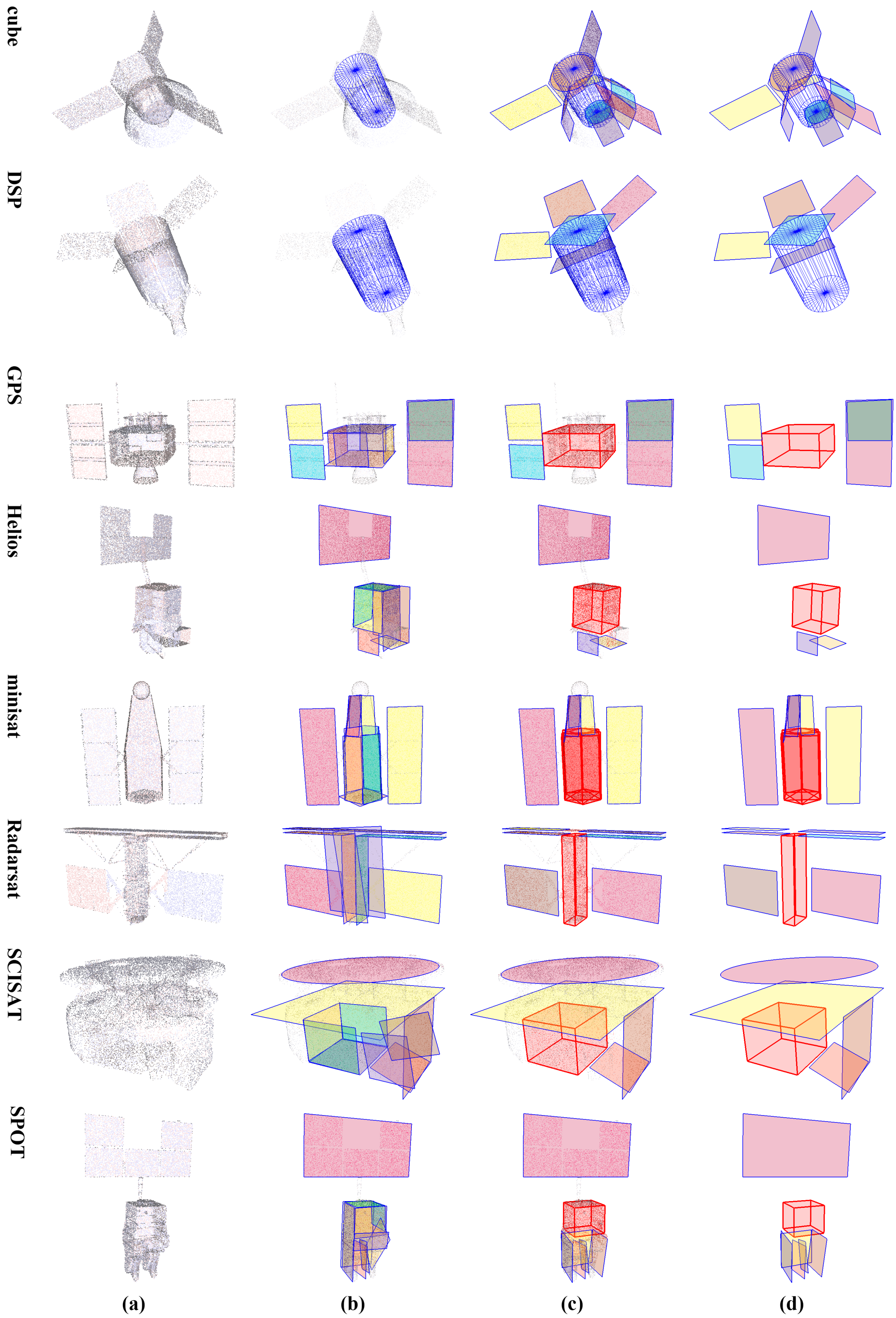

Figure S1. Detection results of the synthesized point cloud data *20K_00U_00D*. From left to right: (a) the origin input point clouds; (b) results of cylinder detection or patch detection after detection of cylinders (the cylinders are rendered in blue, and patches are rendered in different colors.); (c) final results (the detected cuboids are rendered as red boxes.); (d) a clear view of the detected components.

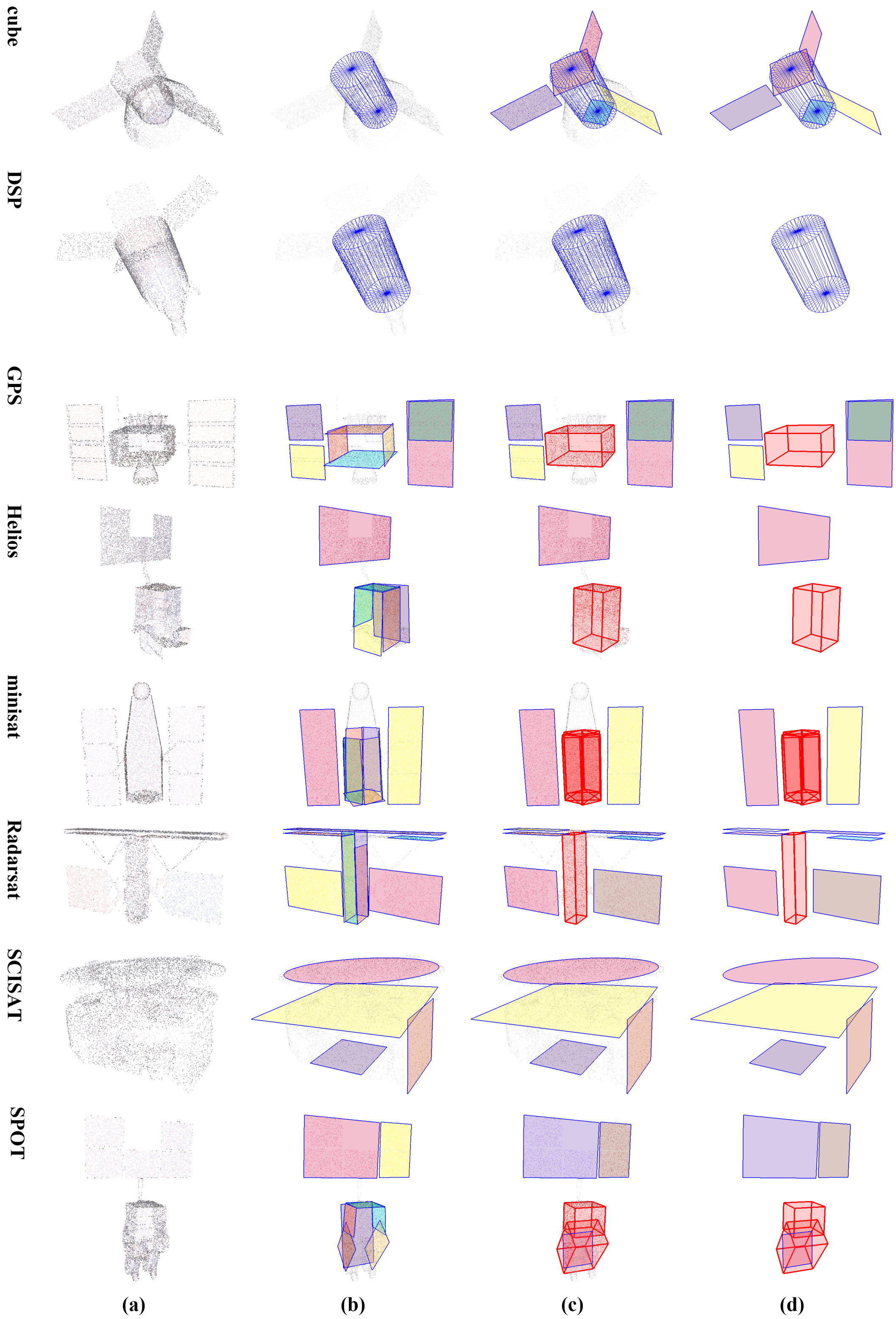

Figure S2. Detection results of the synthesized point cloud data *10K_00U_00D*. From left to right: (a) the origin input point clouds; (b) results of cylinder detection or patch detection after detection of cylinders (the cylinders are rendered in blue, and patches are rendered in different colors.); (c) final results (the detected cuboids are rendered as red boxes.); (d) a clear view of the detected components.

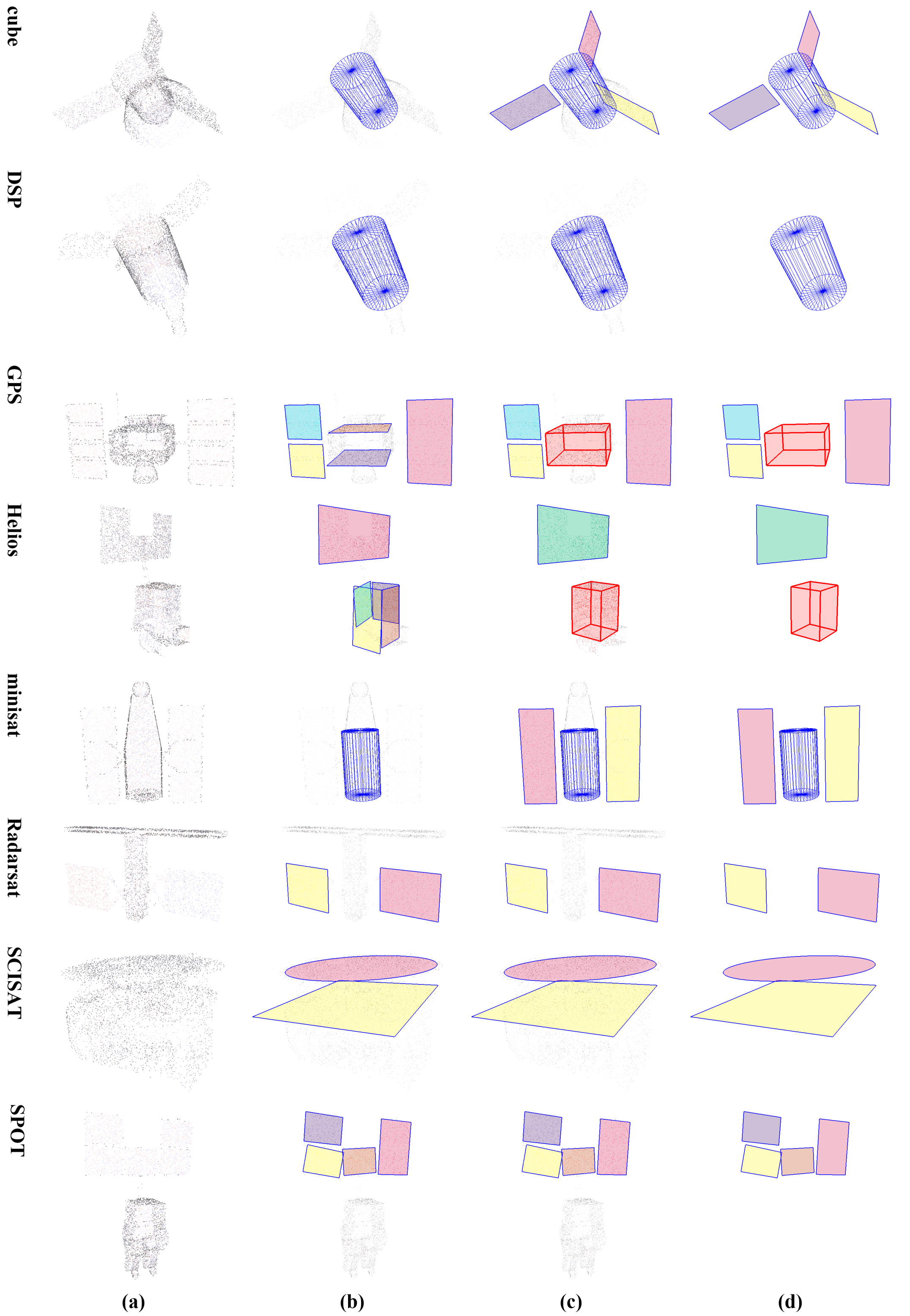

Figure S3. Detection results of the synthesized point cloud data *05K_00U_00D*. From left to right: (a) the origin input point clouds; (b) results of cylinder detection or patch detection after detection of cylinders (the cylinders are rendered in blue, and patches are rendered in different colors.); (c) final results (the detected cuboids are rendered as red boxes.); (d) a clear view of the detected components.

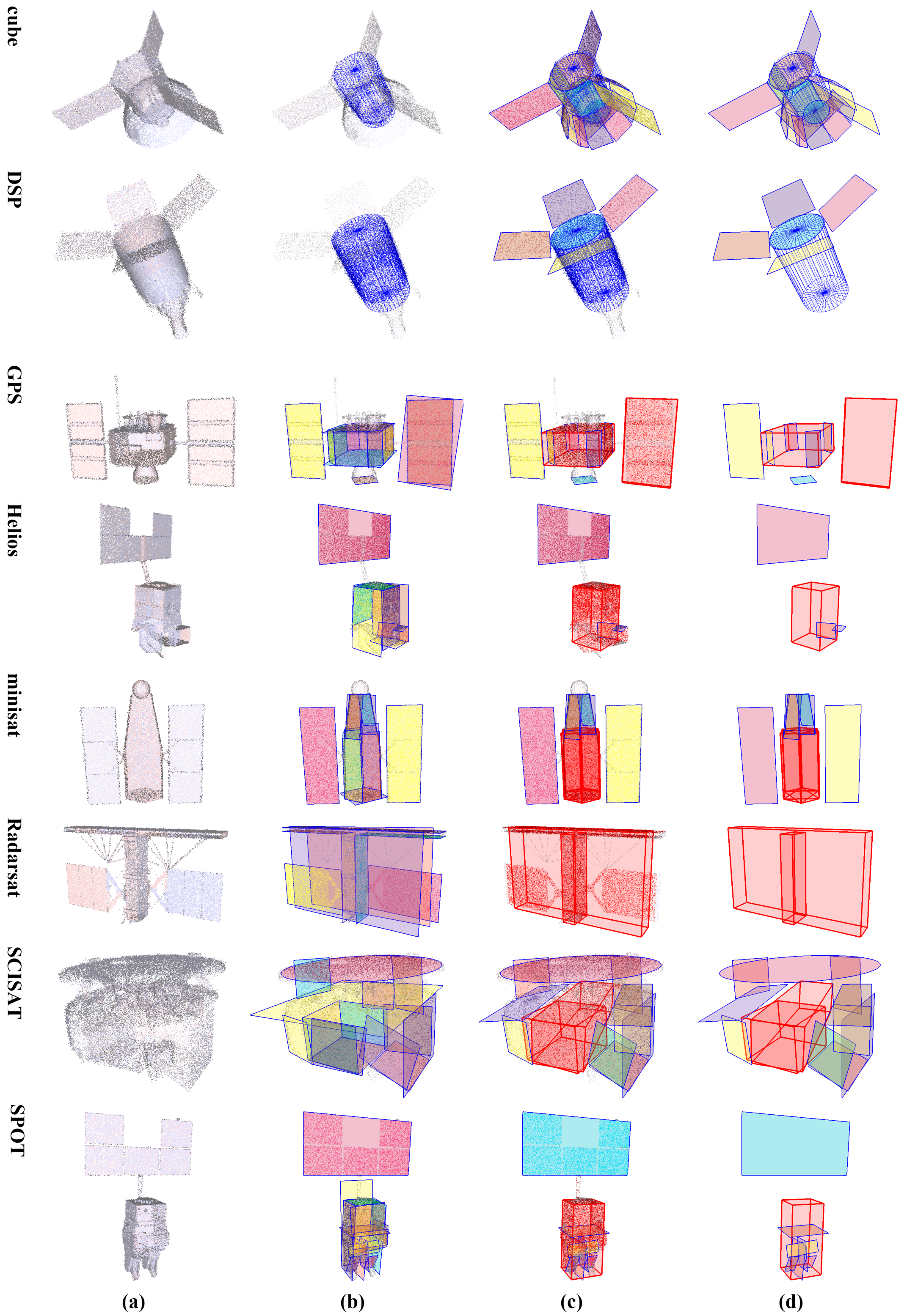

Figure S4. Detection results of the synthesized point cloud data *50K_01U_00D*. From left to right: (a) the origin input point clouds; (b) results of cylinder detection or patch detection after detection of cylinders (the cylinders are rendered in blue, and patches are rendered in different colors.); (c) final results (the detected cuboids are rendered as red boxes.); (d) a clear view of the detected components.

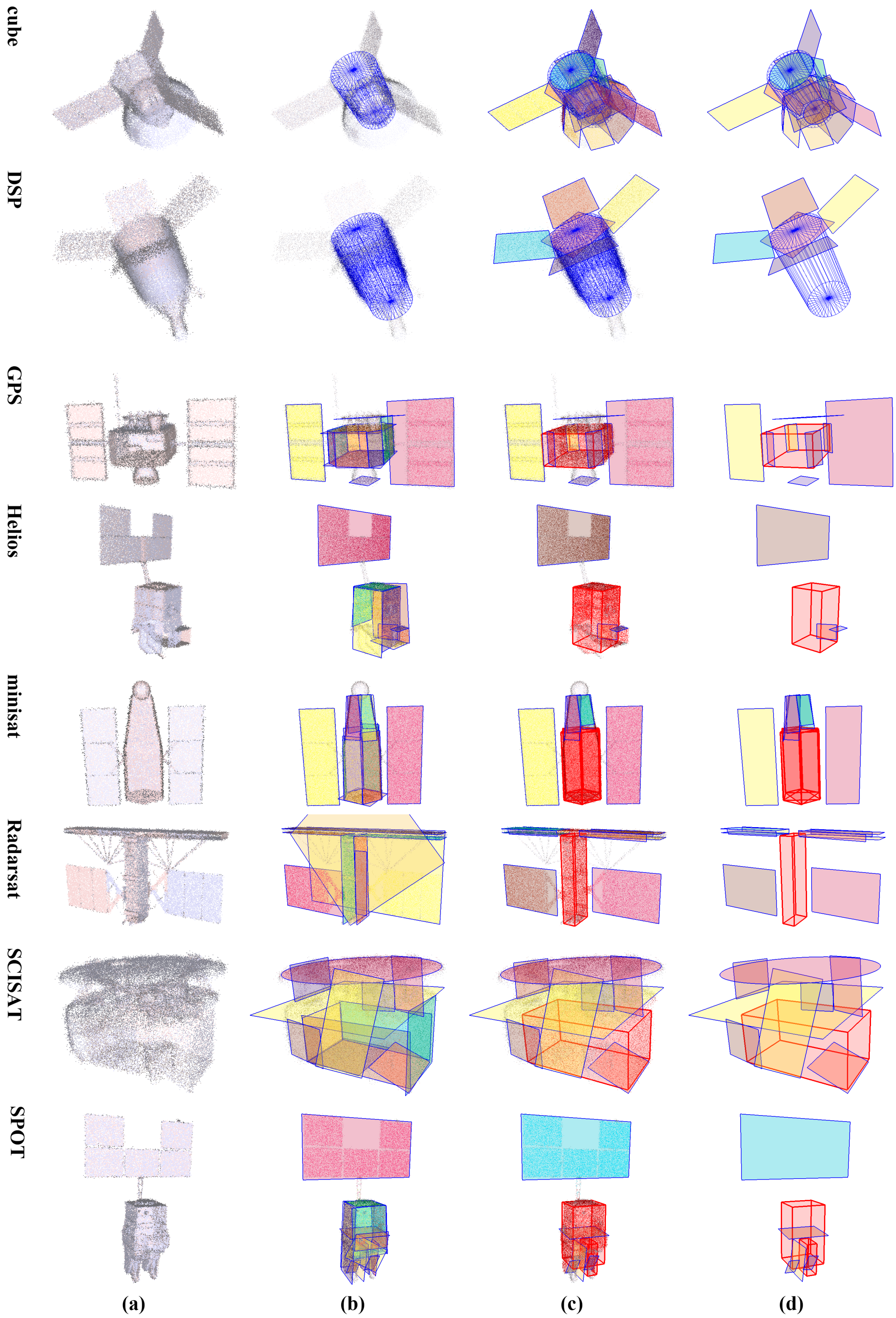

Figure S5. Detection results of the synthesized point cloud data *50K_02U_00D*. From left to right: (a) the origin input point clouds; (b) results of cylinder detection or patch detection after detection of cylinders (the cylinders are rendered in blue, and patches are rendered in different colors.); (c) final results (the detected cuboids are rendered as red boxes.); (d) a clear view of the detected components.

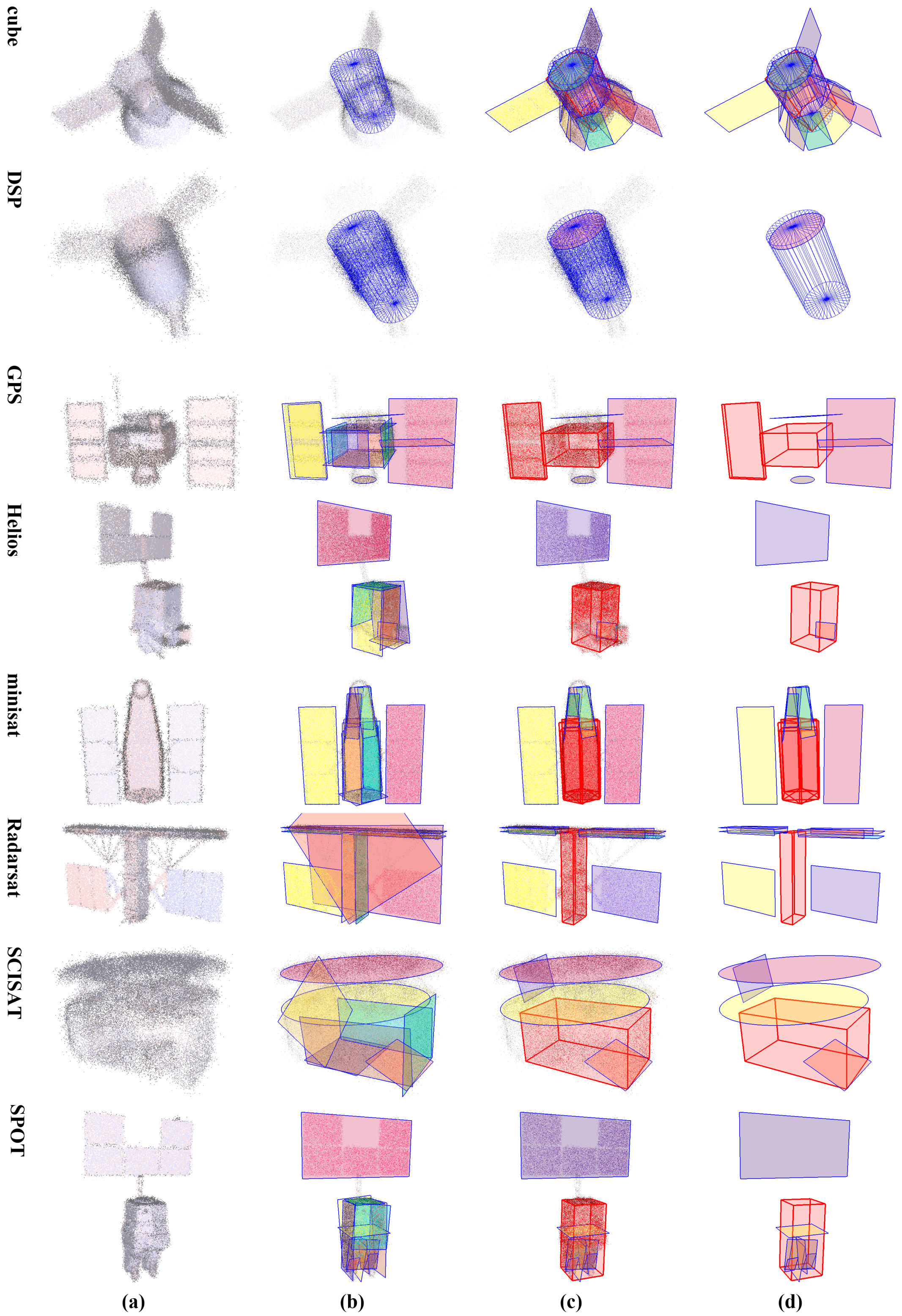

Figure S6. Detection results of the synthesized point cloud data *50K_04U_00D*. From left to right: (a) the origin input point clouds; (b) results of cylinder detection or patch detection after detection of cylinders (the cylinders are rendered in blue, and patches are rendered in different colors.); (c) final results (the detected cuboids are rendered as red boxes.); (d) a clear view of the detected components.

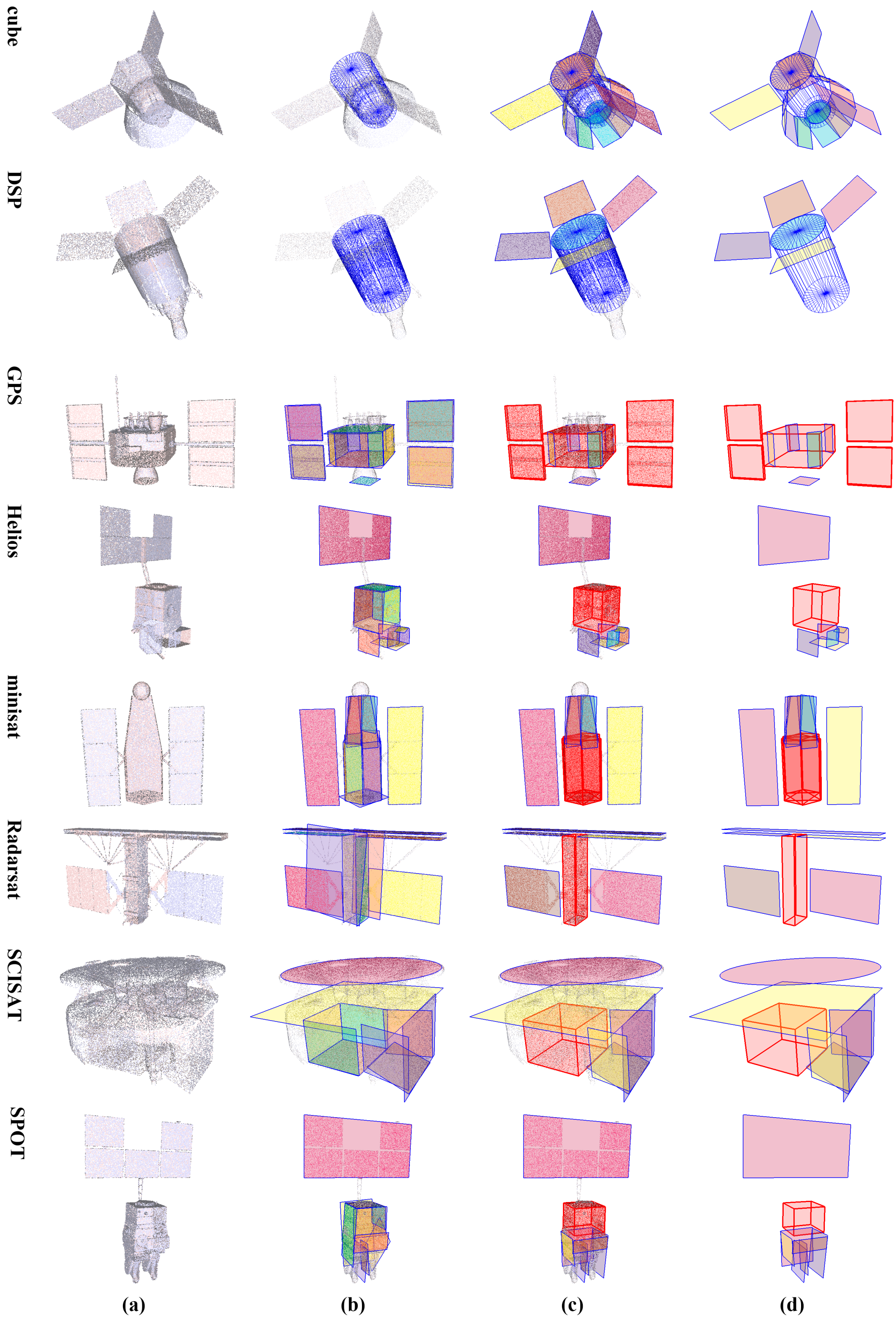

Figure S7. Detection results of the synthesized point cloud data *50K_00U_05D*. From left to right: (a) the origin input point clouds; (b) results of cylinder detection or patch detection after detection of cylinders (the cylinders are rendered in blue, and patches are rendered in different colors.); (c) final results (the detected cuboids are rendered as red boxes.); (d) a clear view of the detected components.

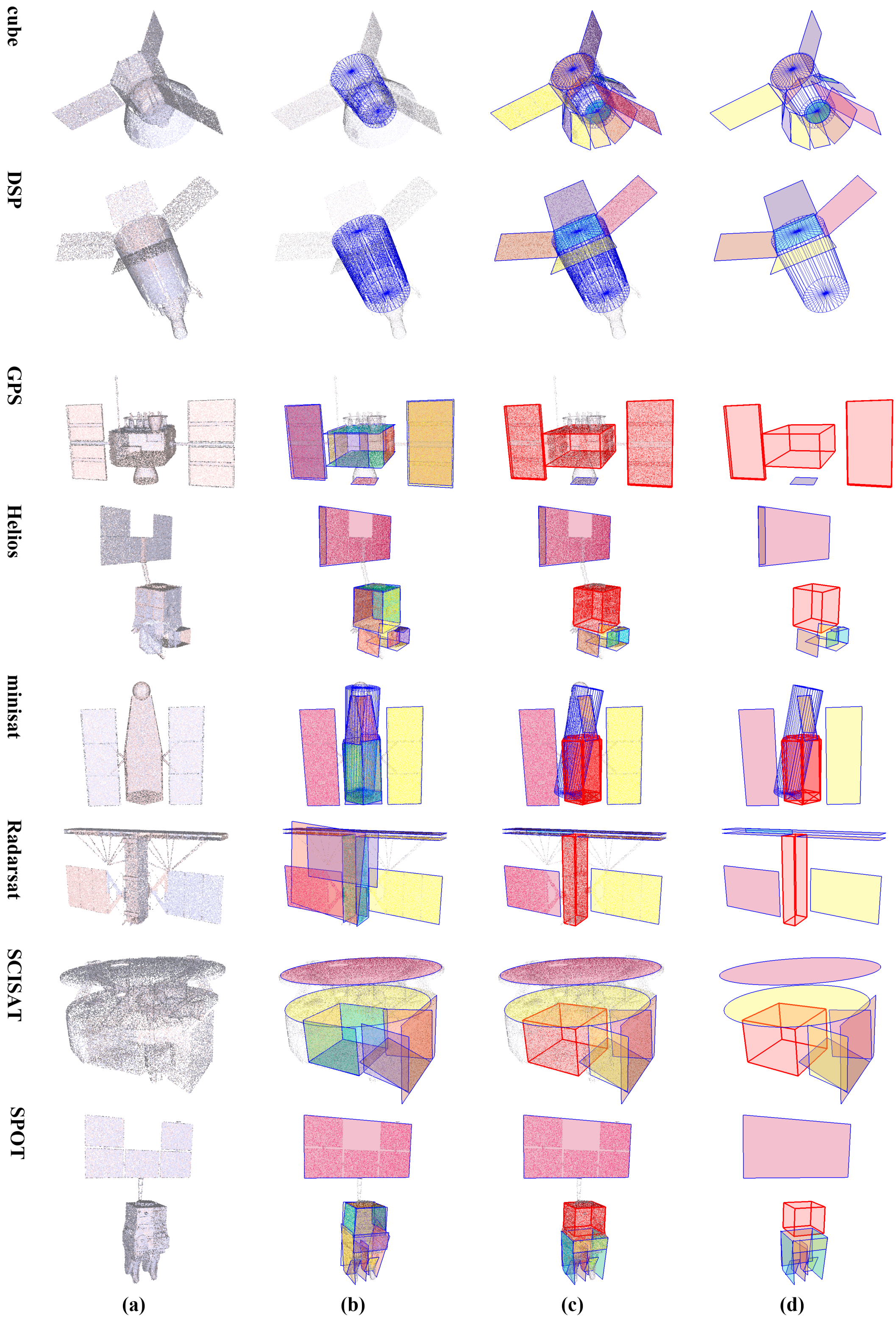

Figure S8. Detection results of the synthesized point cloud data *50K_00U_10D*. From left to right: (a) the origin input point clouds; (b) results of cylinder detection or patch detection after detection of cylinders (the cylinders are rendered in blue, and patches are rendered in different colors.); (c) final results (the detected cuboids are rendered as red boxes.); (d) a clear view of the detected components.

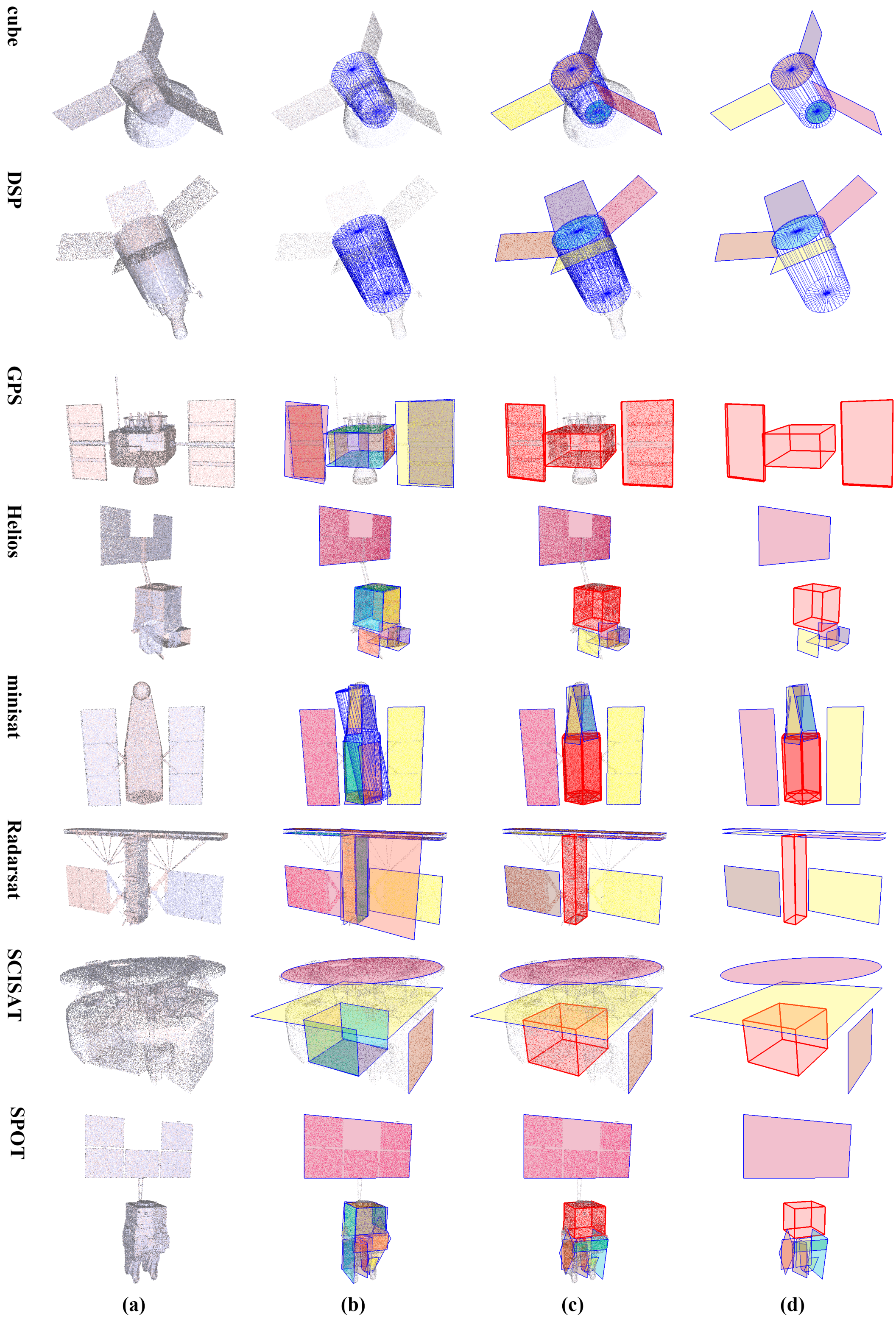

Figure S9. Detection results of the synthesized point cloud data *50K_00U_15D*. From left to right: (a) the origin input point clouds; (b) results of cylinder detection or patch detection after detection of cylinders (the cylinders are rendered in blue, and patches are rendered in different colors.); (c) final results (the detected cuboids are rendered as red boxes.); (d) a clear view of the detected components.
